# The Nutrition and Enjoyable Activity for Teen Girls (NEAT girls) randomized controlled trial for adolescent girls from disadvantaged secondary schools: rationale, study protocol, and baseline results

**DOI:** 10.1186/1471-2458-10-652

**Published:** 2010-10-28

**Authors:** David R Lubans, Philip J Morgan, Deborah Dewar, Clare E Collins, Ronald C Plotnikoff, Anthony D Okely, Marijka J Batterham, Tara Finn, Robin Callister

**Affiliations:** 1 School of Education, University of Newcastle, Newcastle, Callaghan Campus, Australia; 2 School of Health Sciences, University of Newcastle, Newcastle, Callaghan Campus, Australia; 3 Interdisciplinary Educational Research Institute, University of Wollongong, Wollongong, Australia; 4 Centre for Statistical and Survey Methodology, University of Wollongong, Wollongong, Australia; 5 School of Biomedical Sciences, University of Newcastle, Newcastle, Callaghan Campus, Australia

## Abstract

**Background:**

Child and adolescent obesity predisposes individuals to an increased risk of morbidity and mortality from a range of lifestyle diseases. Although there is some evidence to suggest that rates of pediatric obesity have leveled off in recent years, this has not been the case among youth from low socioeconomic backgrounds. The purpose of this paper is to report the rationale, study design and baseline findings of a school-based obesity prevention program for low-active adolescent girls from disadvantaged secondary schools.

**Methods/Design:**

The Nutrition and Enjoyable Activity for Teen Girls (NEAT Girls) intervention will be evaluated using a group randomized controlled trial. NEAT Girls is a 12-month multi-component school-based intervention developed in reference to Social Cognitive Theory and includes enhanced school sport sessions, interactive seminars, nutrition workshops, lunch-time physical activity (PA) sessions, PA and nutrition handbooks, parent newsletters, pedometers for self-monitoring and text messaging for social support. The following variables were assessed at baseline and will be completed again at 12- and 24-months: adiposity, objectively measured PA, muscular fitness, time spent in sedentary behaviors, dietary intake, PA and nutrition social-cognitive mediators, physical self-perception and global self-esteem. Statistical analyses will follow intention-to-treat principles and hypothesized mediators of PA and nutrition behavior change will be explored.

**Discussion:**

NEAT Girls is an innovative intervention targeting low-active girls using evidence-based behavior change strategies and nutrition and PA messages and has the potential to prevent unhealthy weight gain and reduce the decline in physical activity and poor dietary habits associated with low socio-economic status. Few studies have reported the long-term effects of school-based obesity prevention programs and the current study has the potential to make an important contribution to the field.

**Trial registration:**

Australian New Zealand Clinical Trials Registry No: ACTRN12610000330044

## Background

Obesity is a serious health issue and predisposes individuals to an increased risk of morbidity and mortality from conditions such as Type II diabetes, coronary heart disease (CHD), hypertension, hyperlipidemia, and certain cancers [1]. Over the last 20 years the rates of obesity have tripled in developing countries largely due to decreased physical activity (PA) and increased consumption of energy dense foods [2]. Although there is some evidence to suggest that rates of pediatric obesity in developed countries have leveled off in recent years [3], this has not been the case among youth from low socioeconomic backgrounds [4-6]. There is a strong socioeconomic gradient in the prevalence of overweight and obesity among youth from developed countries [4-7] and youth attending schools in disadvantaged areas may be disproportionately susceptible to obesity development [8]. A recent nationally representative sample of US adolescents found that school socioeconomic status (SES) was negatively associated with weight status, even after controlling for individual level SES [8].

The treatment of obese youth is a costly and challenging endeavor and prevention strategies are clearly warranted [9,10]. Schools have been a popular setting for the implementation of interventions to prevent obesity as they have continuous contact with students and the necessary personnel, curriculum and facilities to promote PA and healthy eating [11]. A number of small- and large-scale school-based interventions have been evaluated with varying degrees of success in terms of obesity prevention [11,12]. School-based interventions designed to promote PA and healthy eating can be broadly classified as whole-of-school or targeted. Whole-of-school approaches are not directed toward specific individuals and typically involve changes to the school environment, physical education and/or relevant PA and nutrition (e.g. canteen) policies across the entire school population. For example, the Middle School Physical Activity and Nutrition (M-SPAN) intervention, which involved 24 schools and was guided by a socio-ecological model and incorporated policy and environmental changes [13]. M-SPAN was successful in producing a greater reduction in self-reported BMI among intervention group boys, but not among girls. More recently, Simon and colleagues [14] reported successful obesity prevention following the implementation of the Intervention Centered on Adolescents' Physical activity and Sedentary behavior (ICAPS). Interestingly, ICAPS was a multilevel school-based obesity prevention intervention focused only on the promotion of PA and did not have a nutrition component to promote healthy eating. However, in their review of school-based interventions to treat and prevent obesity, Katz and colleagues [12] suggested that the nutrition component of interventions appeared to be more important for weight reduction than the PA component.

Targeted interventions can involve multiple components, but are directed at specific individuals or groups of individuals. Although the whole-of-school approach has the potential to have a positive impact on the health behaviors of a large number of students, this type of intervention may be less effective among those most in need, such as low-active or overweight students [11,15]. As certain groups are disproportionately affected by obesity, there has been a call for interventions to be targeted within the school towards specific groups of students and be differentiated on the grounds of gender, age and SES [16]. For example, PA declines during adolescence [17,18] especially among adolescent girls from disadvantaged backgrounds, placing this group at an even greater risk of obesity [5,18].

An advantage of using the targeted approach is that interventions can be tailored to the characteristics of specific groups. A number of school-based interventions have targeted adolescent girls 'at risk' of obesity [19-21]. For example, New Moves was a school-based intervention for secondary school girls who were overweight or 'at risk' for becoming overweight due to low levels of PA [20]. Although the intervention was relatively intensive (i.e. 4 PA sessions/week for 16 weeks plus information sessions), it failed to impact upon weight status over the study period of time. One possible explanation for this finding is that the intervention did not include a parental component. Parents play an important role in the shaping of their children's weight-related food and activity behaviors [22] and may need to be included in multi-level approaches to obesity prevention in youth [23].

This paper provides the rationale, study description and baseline findings from the Nutrition and Enjoyable Activity for Teen Girls (NEAT Girls) program. NEAT Girls is a multi-component school-based intervention that combines a range of evidence-based behavior change strategies to promote PA and healthy eating and prevent obesity among low-active adolescent girls. To the authors' knowledge, NEAT Girls is the first school-based obesity prevention program for Australian adolescent girls from economically disadvantaged secondary schools.

## Methods/Design

### Study design

**Figure 1:**
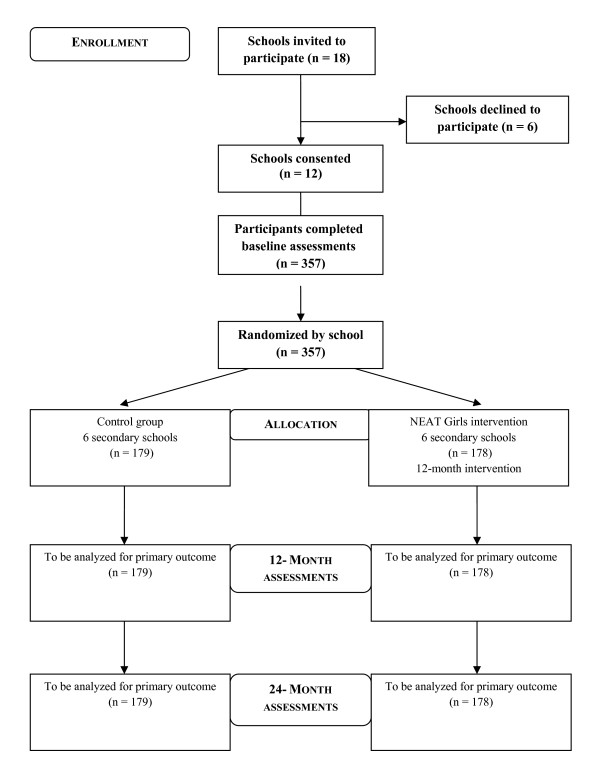
**Study flow**.

NEAT Girls is a group randomized controlled trial (RCT) investigating the effects of a 12-month multi-component school-based PA and nutrition intervention. Assessments took place at baseline (May/June 2010) and will be repeated at 12 months (May/June 2011- end of intervention) and at 24 months (May/June 2012- follow-up)(Figure 1). The design, conduct and reporting of this study will adhere to the Consolidated Standards of Reporting Trials (CONSORT) guidelines [24]. Ethics approval for the study was obtained from the University of Newcastle, Australia and the New South Wales (NSW) Department of Education and Training Human Research Ethics Committees. School principals, parents and study participants provided written informed consent.

### Participants

The Socio-Economic Indexes for Areas (SEIFA) index of relative socioeconomic disadvantage was used to identify eligible secondary schools. The SEIFA index (scale 1 = lowest to 10 = highest) summarizes the characteristics of people and households within an area and is developed using the following data: employment, education, financial well-being, housing stress, overcrowding, home ownership, family support, family breakdown, family type, lack of wealth (no car or telephone), low income, Indigenous status and foreign birth. Government secondary schools located in the Hunter Region and Central Coast areas in NSW, with a SEIFA index of ≤ 5 (bottom 50%) were considered eligible for inclusion. From the 26 eligible secondary schools, 18 schools were contacted and 12 schools were successfully recruited.

Eligible study participants were adolescent girls in Grade 8 (2^nd ^year of secondary school) attending one of the 12 recruited schools. Physical Education (PE) teachers at the study schools identified and recruited participants. To be eligible for the study, students were considered by their teachers to be disengaged in PE and/or not currently participating in organized team or individual sports.

### Sample size calculation

Height and weight assessments were used to calculate body mass index (BMI) which is the primary outcome variable. The primary analysis in this study will be conducted using a linear mixed model. The test of interest will be an F-test with a 1 degree of freedom contrast therefore it was computationally convenient to use the t-test to perform the sample size calculations. The sample size calculation was based on the primary end point of 12 months and does not assume adequate statistical power for the 24 month assessments. The between group difference of 1 kgm^-2 ^was based on the results from a similar trial [25], using a BMI standard deviation of 1.5 kgm^-2 ^[26] and an intraclass correlation coefficient (ICC) of.01 [27]. Variance estimates were adjusted for clustering as proposed by Murray et al [28]. In brief, the standard error of the estimate in the usual t estimation is replaced by $\sqrt{\frac{2({\hat{\sigma }}_{m}^{2}+m{\hat{\sigma }}_{g}^{2})}{mg}}$ where ${\hat{\sigma }}_{m}^{2}$ is the estimate of the unadjusted subject component of the variance, ${\hat{\sigma }}_{g}^{2}$ is the unadjusted school component of the variance, m is the number of subjects per school and g is the number of schools per intervention. For the initial a priori estimate of 24 subjects per school to complete the first year of the study a power of 0.92 was calculated. The minimum sample size recruited was 23, given a 20% dropout, a post hoc power calculation still demonstrates a power of 0.86 to detect the given effect. These calculations are a conservative estimate based on degrees of freedom allowing for a matched design with two covariates.

### Blinding

Baseline assessments were conducted prior to randomization by research assistants (RAs) who were blinded to treatment allocation. Where possible, posttest assessments will also be conducted by RAs blinded to group allocation.

### Randomization

Following baseline assessments, the 12 schools were match paired (i.e. 6 pairs of schools) based on their geographical location, size and demographics. Schools within each pair were then randomized to either the NEAT Girls intervention or a wait list control group by an individual not involved in the research project.

### Intervention

NEAT Girls is a multi-component school-based intervention and includes enhanced school sport sessions, interactive seminars, nutrition workshops, lunch-time physical activities, PA and nutrition handbooks, parent newsletters, pedometers for self-monitoring and text messaging for social support (Table 1). The intervention combines aspects of our previously successful interventions trialed with adolescents [29,30] and was guided by Bandura's Social Cognitive Theory (SCT) [31]. The intervention components were developed using a taxonomy of behavior change strategies [32] and designed to target potential mediators of PA and nutrition behavior change. The intervention is focused on the promotion of low-cost lifetime and lifestyle physical activities and will be delivered over four school terms (i.e., 12 months) at no cost to the school or students. Lifetime physical activities are those that may be easily carried over into adulthood because they generally need only one or two people to participate. Examples include aerobics, jogging, walking, resistance training, swimming and tennis [33]. Lifestyle physical activities are those performed as part of everyday life, such as walking to school and climbing the stairs. In many Australian secondary schools, extra-curricular/co-curricular programs are often delivered off campus and may require payment to participate. Consequently, the cost of school sport activities has been identified as a barrier to participation among some students [34]. The enhanced school sport sessions (60-80 minutes) will be delivered by teachers and involve a range of activities such as elastic tubing resistance training, yoga, Pilates, and Zumba^®^, power walking, skipping choreography and boxing fitness. The sessions are organized into 4-week units and the sequencing of activities is selected by the students. For example, girls may choose Zumba for 4 weeks followed by 4 weeks of Pilates.

**Table 1:** Intervention components, behavior change techniques and targeted constructs

Intervention component	Dose	Description	Behavior change strategy	Targeted construct
1) Enhanced school sport sessions	40 × 90 minutes	School sport sessions will be delivered by teachers and for the first 10 weeks involve an information component (10-15 minutes) and a PA session (75-80 minutes). The information component will address PA and nutrition recommendations, benefits and behavioral strategies. Teacher-directed PA sessions will include a range of lifetime physical activities organized into four week units. Activities will include resistance training using elastic tubing devices, circuit training, boxing style fitness, Zumba^® ^dance, yoga, skipping rope activities, pedometer activities and a mini-Olympics.	* Prompt specific goal setting * Information on consequences * Prompt intention formation * Provide instruction * Barrier identification * General encouragement * Graded tasks	* Outcome expectations * Social support * Self-efficacy * Physical self-perception * Intentions

2) Interactive seminars	3 × 30 minutes	Participants will attend three interactive seminars delivered by members of the research team. Interactive seminars will revise key PA and nutrition recommendations and behavioral strategies to support the student-directed implementation of the lunch-time activities.	* Provide information about behavior health link * Prompt self-monitoring of behaviors * Plan social support or social change * Prompt barrier identification	* Outcome expectations * Social support * Self-efficacy * Intentions

3) Nutrition workshops	3 × 90 minutes	Students will participate in three nutrition workshops delivered by APDs which will provide dietary information and focus on the preparation of inexpensive healthy meals. The activities are planned to develop lifetime nutrition skills that facilitate healthy weight maintenance, including: interpreting nutritional information on food labels, recipe modification and preparation, energy balance and kilojoule concept.	* Information on food and nutrition * Model or demonstrate the behavior * Graded tasks	* Outcome expectations * Self-efficacy * Intentions

4) Lunch-time PA sessions	30 × 30 minutes	Student-directed PA sessions involving a range of lifetime physical activities. These sessions will complement the activities offered in the enhanced school sport sessions. In addition, participants will be encouraged to recruit and instruct Grade 7 students in a range of lifetime physical activities.	* Model or demonstrate the behavior * Graded tasks * Prompt identification as a role model	* Outcome expectations * Social support * Physical self-perception * Self-efficacy

5) PA and nutrition handbooks	10 weeks	Participants will be provided with PA and nutrition handbooks. The handbooks will include 10 weeks of information and home challenges designed to promote PA and healthy eating for parents and participants.	* Provide information about behavior health link * Prompt self-monitoring of behaviors * Plan social support or social change	* Self-efficacy * Social support * Outcome expectations * Intentions

6) Parent newsletters	1 × school term (4 in total)	Parents of study participants will be provided with newsletters describing the study progress and detailing information designed to encourage support of their children's PA and healthy eating behaviors in the home environment.	* Provide feedback on performance * Plan social support or social change * General encouragement * Provide information about behavior health link	* Outcome expectations * Social support * Self-efficacy

7) Pedometers	9 months	Participants will be provided with pedometers and encouraged to initiate goal setting and self-monitoring behaviors. Participants will also be given pedometer challenges to complete over the holiday periods.	* Prompt self-monitoring * Prompt specific goal setting	* Outcome expectations * Social support * Self-efficacy

8) Text messaging	1 × week (40 weeks) 2 × week (10 weeks)	Students will be sent weekly and twice weekly text messages encouraging them to be physically active and eat healthily. Text messages will also provide strategies to overcome barriers to PA and healthy eating. Students without mobile phones will receive these messages via email.	* Plan social support or social change * General encouragement * Provide information about behavior health link * Barrier identification	* Outcome expectations * Social support * Self-efficacy

*Note*. APD = Accredited practicing dietitians; PA = physical activity.

The NEAT Girls intervention is based on 10 key PA and nutrition messages (Table 2). For the first school term, the enhanced school sport sessions include an information component (10-15 minutes) delivered by cooperating teachers from the study schools, that integrates the 10 key health messages. The three interactive seminars will be delivered by members of the research team and will reinforce the NEAT Girls PA and nutrition messages. The weekly lunchtime PA sessions will complement the enhanced school sport sessions and be organized and delivered by the girls participating in the study. The PA and nutrition handbook includes 10 weeks of information and home challenges designed to promote PA and healthy eating among parents and study participants. Three practical nutrition workshops will be delivered in the study schools by Accredited Practicing Dietitians (APDs) and will focus on providing dietary information and strategies designed to develop lifetime nutrition skills that facilitate the maintenance of a healthy weight, e.g. food label reading, recipe modification, energy balance, measuring kilojoules and appropriate portion sizes and the preparation of inexpensive healthy snacks and meals. Participants will be provided with pedometers to encourage PA goal setting and self-monitoring and to use during school holidays for assigned PA challenges. Pedometers will also be used for self-monitoring and to remind participants of the importance of incidental PA and the contribution of non-exercise activity thermogenesis to weight control [35]. Study newsletters will be sent to parents at four time periods over the 12-month intervention. The newsletters will describe program content and study progress, including participants' time spent in sedentary behaviors and PA and fruit and vegetable consumption from baseline assessments. This information is designed to raise awareness and encourage parents to support their children's PA and healthy eating behaviors. Text messaging has emerged as a potential tool for improving health in youth [36] and students will be sent weekly (Terms 2/3 and during the school holidays) and bi-weekly (Term 4) text messages encouraging them to be physically active and eat healthily. The text messages do not require a response from the students and will be both motivational and informational. Messages will be sent at a time appropriate for the specific message. For example, messages to encourage PA will be sent in the critical window immediately after school.

**Table 2:** NEAT Girls physical activity and nutrition messages

Study week	Physical activity and nutrition message
Week 1	Be active in any way you can
Week 2	Aim to eat fruit and vegetables every day
Week 3	Be active with friends and family
Week 4	Eat a healthy breakfast every day
Week 5	Reduce your sitting time during school lunch breaks, after school and on the weekends
Week 6	Monitor your portion sizes during dinner and eat at the dinner table
Week 7	Identify excuses for not being active
Week 8	Drink more water and swap sugary drinks for sugar-free drinks
Week 9	Keep track of your physical activity using a pedometer diary
Week 10	Reduce your junk food snacks

The NEAT Girls intervention includes a reward system to encourage study compliance with bronze, silver and gold awards available at each school. Certificates are made available to all participants satisfying the eligibility criteria and in addition, girls who achieve silver and gold awards will enter a draw to win $30 and $40 gift certificates, respectively. To be eligible for the bronze certificate, participants must attend at least 80% of enhanced school sport sessions in any one term and complete at least 50% of the home challenges in their PA and nutrition handbook or attend at least 50% of lunchtime sessions in any one term. To attain the silver award, participants must attend at least 80% of enhanced school sport sessions for any two terms and at least 50% of lunchtime sessions in any two terms, facilitate at least one PA session and complete at least 50% of the home challenges. Finally, to achieve a gold award, participants must attend at least 80% of enhanced school sport sessions for all 4 terms, complete at least 7/10 of the home challenges, attend at least 70% of lunchtime sessions and schedule at least two lunchtime sessions.

To facilitate the implementation of the NEAT Girls program, cooperating teachers will be invited to attend two one day workshops at the university. The workshops will be designed to help teachers deliver the intervention components and findings from the baseline assessments will also be reported back to the teachers. All intervention schools have been provided with a standard equipment pack (value = $US1300), which consists of the following: 15 Gymsticks (elastic tubing resistance training devices), 8 sets of boxing gloves, 4 sets of focus pads, 2 large skipping ropes, 12 single skipping ropes, iPod docking station, Zumba^® ^fitness DVD, Yoga DVD, Pilates DVD, skipping choreography DVD,15 fit balls, 2 giant beach balls, pedometers (1 for each student), recipe cards and TEMplates(tm) (1 for each student). The TEMplate(tm) is a portion disc that fits on a dinner plate to guide selection of appropriate portion sizes of a variety of vegetables, lean protein sources and healthy carbohydrates.

Following the completion of 24-month assessments the control schools will receive the equipment packs and intervention materials. A condensed version of the NEAT Girls intervention will be offered to the schools at this time.

### Outcomes

Baseline assessments were conducted by trained RAs at the study schools. A protocol manual was used by RAs which included specific instructions for conducting all assessments. Physical assessments were conducted in a sensitive manner (e.g. weight was measured away from other students) and questionnaires were completed after the physical assessments in exam-like conditions. The primary outcome measure is BMI; secondary outcomes measures are body fat from bioelectrical impedance, muscular fitness, objectively measured PA, dietary and sedentary behaviors and physical self-perception.

#### Height and weight

Weight was measured in light clothing without shoes using a portable digital scale (Model no. UC-321PC, A&D Company Ltd, Tokyo Japan) to the nearest 0.1 kg. Height was recorded to the nearest 0.1 cm using a portable stadiometer (Model no. PE087, Mentone Educational Centre, Australia). Body mass index (BMI) was calculated using the equation (weight[kg]/height[m]^2^) and BMI-z scores were calculated using the 'LMS' method [37].

#### Body fat

Percentage body fat, fat mass (FM) and fat free mass (FFM) were determined using the Imp(tm) SFB7 bioelectrical impedance analyzer, which is a multi-frequency, tetra polar bioelectrical impedance spectroscopy device [38].

#### Muscular fitness

A modified version of the 90° push-up test (90PU) was used as a measure of upper body muscular endurance [39]. Participants started in the push-up position with their hands and knees touching the floor and the arms at shoulder width apart. Keeping their back straight and hips extended, participants then lowered themselves to the ground until there was a 90-degree angle at the elbows, with upper arms parallel to the floor. A tester held their hand at the point of the 90-degree angle so that the shoulder of the participant touched the observer's hand, then pushed back up. The push-ups were completed in time to a metronome set at 40 beats per minute with one complete push-up every three seconds. The prone support test was used to provide a measure of core abdominal isometric muscular endurance. Participants started lying face down on a gym mat, then propped themselves up on their elbows and toes so that their body was in a straight line (hips and knees are not to be flexed). The participant was timed to see how long they could hold the prone support position before dropping a knee to the ground.

#### Physical activity

Actigraph accelerometers (MTI models 7164, GT1M and GT3X) were worn by participants during waking hours for seven consecutive days, except while bathing and swimming. The accelerometers were worn on a small elastic belt and positioned in front of the right hip. Trained RAs, following standardized accelerometer protocols [40] fitted the monitors and explained the monitoring procedures to study participants. To improve compliance with the study protocols, participants were sent text messages each morning during the seven-day monitoring period to remind wear. Data were collected and stored in 30-second epochs and the mean activity counts per minute (CPM) was calculated. Age and gender specific cut-points were used to categorize PA into sedentary, light, moderate and vigorous intensity activity [41].

#### Dietary behavior

Dietary intake was assessed using the Australian Eating Survey (AES). AES is a 135-item semi-quantitative food-frequency questionnaire (FFQ) which was previously tested for reliability and relative validity [42]. The tool demonstrated acceptable accuracy for ranking nutrient intakes in Australian youth 9 to 16 years [43] and is currently being validated in adults. Portion sizes for individual food items were accessed from the Australian Bureau of Statistics (ABS) and unpublished data from the 1995 Australian National Nutrition Survey or the "natural" serving size for common items such as a slice of bread. Subjects were asked about frequency of their consumption over the previous 6 months. The frequency options ranged from 'Never' to '4 or more times per day' but varied depending on the food item.

Twenty-one questions relate directly to the intake of vegetables and 11 to fruit, with seasonal availability of some fruits considered in the nutrient analysis. The frequency categories were listed as for other food items, with the question, "When the following fruit is in season, how often do you usually eat it?" with seasonal availability determined by data provided by fresh food markets in Sydney, NSW, in addition to referring to supermarket literature that indicated the months of the year different seasonal fruit was available. The AES includes additional questions about the total number of daily serves of fruit, vegetables, bread, dairy products, eggs, fat spreads, sweetened beverages and snack foods, as well as asking the type of bread, dairy products and fat spreads used. Twelve questions relate to food-related behaviors, including items on frequency of take-away food consumption and eating while watching television.

#### Sedentary behaviors

The Adolescent Sedentary Activity Questionnaire (ASAQ) was used to provide a self-report of time spent in sedentary behaviors [44]. The ASAQ requires respondents to report time spent in the following activities: watching television/videos/DVDs, computers, e-games and e-communication, study, reading, sitting with friends, telephone use, listening or playing music, motorized travel, hobbies and crafts, all performed out of school hours.

#### Physical activity and dietary social-cognitive mediators

Social cognitive scales for PA (Table 3) and dietary (Table 4) behaviors based on constructs from SCT [45] were designed for the study. Participants completed separate PA and nutrition scales for the following constructs: self-efficacy, social support, environmental perceptions (situation), behavioral strategies (self-control), outcome expectations (perceived benefits), outcome expectancies (value placed on benefits) and intentions. The validity and two week test-retest reliability of the measures was assessed in a sample (N = 171) of Australian adolescents (66 = males; 105 = females; mean age = 13.6 ± 1.2 years). Selected subscales (i.e., strength, body fat, appearance, general physical self-concept and global self-esteem) from Marsh's Physical Self-Description Questionnaire [46] were included as potential outcomes and mediators of PA behavior.

**Table 3:** Physical activity mediator scales

Variables	Description and example items	Range (No. of items)	ICC (95% CI)	α	χ^2^	*df*	*p*	GFI	RMSEA
*Physical activity mediators*									
Self-efficacy	Confidence in ability to adopt, maintain and overcome barriers to PA behaviors. For example: *"I can still find the time to be physically active even when I've had a busy day"*.	1-6 (5)	0.91 (0.88 to 0.93)	0.69	3.82	5	0.58	0.99	0.00
Environmental perceptions	An individual's mental representation of their environment that may influence their PA behavior:								
	• Home environment - For example: *"At home I have access to equipment that helps me to be physically active - e.g. bikes, balls, skateboards"*.	1-6 (3)	0.88 (0.83 to 0.91)	0.63	11.22	8	0.19	0.98	0.05
	• School environment - For example: *"At school, facilities are available during recess/lunch for me to be physically active - e.g. the gym, dance studio, sports equipment".*	1-6 (3)	0.85 (0.79 to 0.89)	0.65	11.22	8	0.19	0.98	0.05
Social support	Social influences that reinforce PA through encouragement and role modeling:								
	• Peer support - *"...how often did you make plans with your friends to be physically active together?'*	1-5 (4)	0.91 (0.88 to 0.94)	0.78	27.40	19	0.10	0.97	0.05
	• Family support - For example: *"... how often did members of your family participate in physical activities/sports with you?"*	1-5 (4)	0.86 (0.81 to 0.90)	0.74	27.40	19	0.10	0.97	0.05
Behavioral strategies	Self-reinforcement for PA achieved through setting goals, monitoring behavior and self-reward. For example: *"...did you organize to be physically active with a friend or family member?"*	1-5 (6)	0.91 (0.88 to 0.93)	0.79	15.45	9	0.16	0.97	0.07
Outcome expectations	Anticipated outcomes of PA such as the benefits. For example: *"Participation in regular physical activity can help me to improve my fitness".*	1-6 (5)	0.82 (0.75 to 0.86)	0.75	11.26	5	0.03	0.97	0.09
Outcome expectancies	The value placed on anticipated outcomes of PA. For example: *"How important is improving your fitness to you?"*	1-4 (5)	0.88 (0.83 to 0.91)	0.66	15.74	5	0.01	0.97	0.11
Intentions	Intention to be physically active.	1-4 (1)	0.79 (0.72 to 0.85)	NR	NR	NR	NR	NR	NR

*Note*. *ICC *= Intra class correlation for 2-week test-retest reliability; α = Cronbach alpha; χ^2 ^= Chi-square; *df *= degrees of freedom; *p *= probability; GFI = goodness of fit index; RMSEA = root mean square of approximation; NR = Not relevant.

**Table 4:** Dietary social-cognitive mediator scales

Variables	Description and example items	Range (No. of items)	ICC (95% CI)	α	χ^2^	*df*	*p*	GFI	RMSEA
*Dietary mediators*									
Self-efficacy	Confidence in ability to adopt, maintain and overcome barriers to healthy eating behaviors. For example: *"I find it easy to choose a healthy snack when I eat between meals - e.g. fruit, reduced fat yoghurt"*.	6	0.89 (0.85 to 0.92)	0.70	17.41	9	0.04	0.97	0.07
									
Environmental perceptions	An individual's mental representation of their environment that may influence their dietary behaviors. For example: *"At home, fruit is always available to eat - e.g. fresh, canned or dried".*	4	0.81 (0.75 to 0.86)	0.79	0.90	2	0.64	1.00	0.00
Social support	Social influences that reinforce healthy eating through encouragement and role modeling. For example: *"...how often do your parents prepare a healthy home-cooked dinner for you?"*	6	0.89 (0.85 to 0.92)	0.68	10.24	9	0.33	0.98	0.03
Behavioral strategies	Self-reinforcement for healthy eating achieved through setting goals, monitoring behavior and self-reward. For example: *"During meals do you leave food on your plate once you feel full?"*	6	0.88 (0.84 to 0.91)	0.75	6.69	9	0.67	0.99	0.00
Outcome expectations	Anticipated outcomes of healthy eating such as the benefits. For example: *"Healthy eating can help me to control my weight".*	5	0.84 (0.79 to 0.88)	0.72	14.67	5	0.01	0.97	0.10
Outcome expectancies	The value placed on anticipated outcomes of healthy eating. For example: *"How important is controlling your weight to you?"*	5	0.89 (0.87 to 0.92)	0.65	4.10	5	0.54	0.99	0.00
Intentions	Intention to eat healthily. For example: *"... do you intend to choose reduced-fat foods and drinks whenever you have a choice?"*	6	0.83 (0.77 to 0.87)	0.71	9.77	5	0.08	0.98	0.08

*Note*. PA = physical activity; ICC = Intra class correlation for 2-week test-retest reliability; α = Cronbach alpha from the study sample; χ^2 ^= Chi-square; *df *= degrees of freedom; *p *= probability; GFI = goodness of fit index; RMSEA = root mean square of approximation.

#### Process evaluation

A detailed process evaluation will be undertaken to assess the feasibility of the NEAT Girls program. This will include recruitment (achievement of target sample size), retention (retention rates at 12- and 24-month follow-ups), attendance (at enhanced school sport, interactive seminars and lunch-time physical activities), intervention fidelity (24 randomly selected sessions will be observed by a member of the research team and participants will submit their PA and nutrition handbooks), acceptability and program satisfaction (students and teachers will complete detailed process evaluation questionnaires at the completion of the study).

### Statistical methods

Statistical analyses will be conducted using mixed models which have the advantage of being robust to the biases of missing data and provide appropriate balance of Type 1 and Type 2 errors [47]. The models will be specified to adjust for the clustered nature of the data and the analysis conducted using established models [48]. The study was designed to randomize in matched pairs, matching has been shown to improve the power if the number of groups per condition is greater than 10 or the matching correlation is greater than 0.30. As this study has less than 10 groups the power analysis and analysis plan have conservatively been designed to incorporate the matching if the correlations between the matching variable and the dependent variable (BMI) or the correlation on BMI between members of a pair are high [49]. Multiple imputation will also be considered as a sensitivity analysis if the dropout rate is substantial. Cohen's *d *[50] will be used to determine effect sizes and will be calculated using the mean difference (12- and 24-months minus baseline) between groups and the pooled standard deviation of change for the whole group. Mediation analysis will be conducted by assessing hypothesized social-cognitive mediators of PA and nutrition behavior change using the PRODuct of Confidence Limits for INdirect effects (PRODCLIN) program [51]. The characteristics of the study participants at baseline are reported in the results section of this paper. Estimates of baseline characteristics for the treatment and control groups were adjusted for clustering by school using PROC MIXED in SAS V 9.1 (SAS Institute Inc, Cary, NC) and intraclass correlation coefficients (ICCs) were calculated for key outcomes. Differences between intervention and control groups at baseline were examined using independent samples t-tests in PASW Statistics 17 (SPSS Inc. Chicago, IL) software and alpha levels were set at *p *< .05.

## Results

Twelve schools were recruited and 357 participants were assessed at baseline, representing a 99.2% of the targeted sample size (Table 5). Results are provided as means ± standard deviations, unless otherwise noted. There were no statistically significant differences between control and intervention groups at baseline for any of the demographic or outcome variables. Most participants were born in Australia (97.8%), spoke English at home (98.6%), and identified their cultural background as Australian (85.4%). A high percentage of participants were classified as overweight (26.1%) and obese (16.8%).

**Table 5:** Baseline characteristics of study sample

Characteristics	Control (*n *= 179)		NEAT Girls (*n *= 178)		Total (*N *= 357)	
	Mean	SD(SE)	Mean	SD(SE)	Mean	SD(SE)

Age (years)	13.2(13.2)	.4(0.04)	13.2(13.2)	.4(0.04)	13.2	.5(0.024)

Country of birth, *n *(%)^1^	174	97.2%	175	98.3%	349	97.8%
Language spoken at home, *n *(%)^2^	176	98.3%	176	98.9%	352	98.6%
Cultural background^3^						
Australian, *n *(%)	153	85.5%	152	85.4%	305	85.4%
Asian, *n *(%)	1	.6%	3	1.7%	4	1.1%
European, *n *(%)	18	10.1%	18	10.1%	36	10.1%
Other, *n *(%)	7	4.0%	4	2.2%	11	3.1%

Weight (kg)	58.4(58.4)	13.8(1.44)	58.4(58.3)	14.2(1.44)	58.4	13.9(0.74)
Height (m)	1.61(1.61)	.07(0.00)	1.60(1.60)	.06(0.01)	1.60	.07(0.00)
BMI (kg/m^2^)	22.6(22.6)	4.5(0.48)	22.7(22.7)	4.7(0.48)	22.6	4.6(0.24)
BMI *z*-score	.78(0.78)	1.17(0.12)	.82(0.82)	1.12(0.12)	.80	1.14(0.06)
BMI Category						
Underweight, *n *(%)	1	.6%	1	.6%	2	.6%
Healthy weight, *n *(%)	99	55.3%	103	57.9%	202	56.6%
Overweight, *n *(%)	50	27.9%	43	24.2%	93	26.1%
Obese, *n *(%)	29	16.2%	31	17.4%	60	16.8%
BIA (body fat %)	28.3(28.3)	6.8(1.02)	29.6(29.5)	6.5(1.03)	28.9	6.7(0.35)

Push-up test (reps)	12(12.1)	8(1.12)	12(12.1)	9(1.12)	12	8(0.44)
Prone support test (s)	48(48.0)	34(5.32)	54(53.4)	34(5.33)	51	34(1.82)

MVPA (mins/day)^4^	33.6(33.7)	16.4(2.63)	36.9(36.6)	17.7(2.63)	35.3	17.1(1.27)
Meeting MVPA guideline, *n *(%)^5^	11	9.6%	13	11.2	24	10.4%

SSR (mins/day)	262.4(262.5)	170.5(18.81)	284(284.5)	162.3(18.90)	273.2	166.6(8.83)
Meeting SSR guidelines, *n *(%)^6^	23	12.8%	17	9.6%	40	11.2%

Dietary intake (kJ/kg/day)	163.4(163.3)	81.3(8.69)	173.0(173.8)	91.4(8.71)	168.2	86.5(4.58)

*Note*. SD = Standard deviation; SE = Standard error; BMI = Body mass index; BIA = Bioelectrical impedance analysis; MVPA = Moderate-to-vigorous physical activity minutes per day; s = Seconds; mins = Minutes; SSR = Small screen recreation.

^1^Participants born in Australia

^2^Participants who speak English at home.

^3^One participant did not report their cultural background.

^4^n = 114 (Control), n = 116 (Intervention); N = 230 participants wore accelerometers for ≥600 minutes on ≥4 day.

^5^Participants meeting the MVPA of ≥60 minutes of MVPA.

^6^Participants not exceeding the SSR recommendations of ≥120 minutes each day.

The ICC values for BMI, BMI z-score, moderate-vigorous physical activity (MVPA) minutes, SSR and kilojoules per kilogram per day (kJ/kg/day) were .03, .03, .09, .04 and .03, respectively. Two hundred and thirty participants wore accelerometers for ≥600 minutes on at least four days. From this number, only 10.4% met the PA recommendations of at least 60 minutes of MVPA each day (averaged across days monitored). A small proportion of participants (11.2%) met the SSR guidelines of less than 2 hours/day and girls reported consuming 168 (± 87) kilojoules per kilogram per day.

## Discussion

To the authors' knowledge this is the first school-based obesity prevention program for Australian adolescent girls from disadvantaged secondary schools. Targeting low-active girls from such schools is important because PA [52] and fruit and vegetable consumption is lower in this group compared to those from mid- and high-SES strata [53]. Consequently, the prevalence of overweight and obesity is significantly higher among adolescents from low-SES backgrounds [4-6].

We successfully recruited 12 secondary schools and 357 adolescent girls. The target sample size was achieved in less than 6-weeks, suggesting that the proposed program was appealing to the target group. The challenge of recruiting adolescents has been noted in the literature and this difficulty may prevent many studies from being conducted and published [54]. PE teachers at the study schools were responsible for the identification and recruitment of low-active girls. Girls were eligible if they were considered by their teachers to be disengaged in PE and/or not currently participating in organized team or individual sports.

Of those participants who wore accelerometers for ≥600 minutes on at least four days, only 10% met the PA recommendations of at least 60 minutes of MVPA each day. Based on a representative sample of US adolescents who had worn accelerometers for at least one day, Troiano and colleagues [55] reported that only 3.4% of girls aged 12-15 years attained sufficient PA to meet public health recommendations. While the percentage of adolescent girls achieving the MVPA guidelines was higher in our study, our prevalence estimates were based on those who wore accelerometers for at least four days. It has been suggested that four days of objective monitoring of PA is required to provide an accurate assessment of habitual activity [56] and estimates based on only one day of monitoring may be less precise than those based on multiple days. In the current study, participants were sent text messages every morning to remind them to wear their pedometers. This strategy contributed to a high level of compliance among participants, but a number of accelerometers malfunctioned, resulting in unusable data for 50 participants.

Forty-three percent of the study sample were overweight (26.1%) or obese (16.8%). This represents almost double the prevalence of overweight and obesity (23%) found in the Australian National Children's Nutrition and Physical Activity Survey [57]. The high prevalence of overweight and obesity found in the study sample is consistent with previous studies which have identified a higher prevalence of obesity in adolescents from low-SES groups [4-6]. An advantage of our recruitment strategy is that we were able to identify and attract adolescent girls to a targeted school-based program without stigmatization and our process measures will determine whether girls' participation in the study was a positive experience.

Preventing unhealthy weight gain among adolescent girls is difficult, and many school-based interventions, both targeted [19-21,58] and whole-of-school [13,59], have been unsuccessful. One possible reason for the failure of previous approaches is that they have failed to impact upon family support for PA and healthy eating. In a two year study with Belgian adolescents, Haerens and colleagues [60] found that their intervention with parental support was successful in preventing unhealthy weight gain in girls. Engaging parents in health promotion programs is difficult [61], but their influence on PA and dietary behaviors is pervasive and therefore strategies to involve parents are warranted [23]. The NEAT Girls intervention includes a number of innovative strategies designed to encourage parents to support their daughters' PA and dietary behaviors.

The NEAT Girls intervention has the potential to be a successful and sustainable approach to obesity prevention in adolescent girls from disadvantaged schools. It has a strong theoretical foundation [31] and the intervention strategies were designed to target specific mediators of PA and nutrition behavior change. To improve our understanding of behavior change, hypothesized mediators will be tested in a mediating variable framework [62,63]. To help reduce the decline in PA associated with adolescence [18], participants will be provided with a range recreational lifetime physical activities that can be easily completed within and beyond the school setting. Furthermore, the majority of the intervention will take place during timetabled school sport and the lunch-time physical activities will be delivered by study participants, both of which will contribute to the sustainability of the program.

In terms of study adherence, participants will be provided with pedometers for self-monitoring and encouraged to increase their incidental PA. Adherence is inversely related to exercise intensity and interventions promoting moderate intensity lifestyle activity have been found to have good adherence [30,64]. NEAT Girls includes a number of components designed to support PA and dietary behavior change, including rewards, text messaging, nutrition workshops and strategies to engage parents to support the PA and dietary behaviors of their children. These methods will help to increase motivation and reduce the decline in PA associated with adolescence.

## Conclusion

NEAT Girls is an innovative intervention targeting low-active girls using evidence-based behavior change strategies and has the potential to prevent unhealthy weight gain and reduce the decline in PA associated with adolescence. Few studies have reported the effects of mediation analyses from youth interventions to promote PA and healthy eating [65,66] and this study will provide insights into the mechanisms of behavior change to assist in the design of future obesity prevention trials. These insights will build on the existing knowledge and help to guide interventions targeted towards 'at risk' groups.

## Competing interests

The authors declare that they have no competing interests.

## Authors' contributions

DRL, PJM, RC, CEC, ADO and RCP obtained funding for the research. All authors contributed to developing the protocols and reviewing, editing, and approving the final version of the paper. DRL, DD, PJM, CEC, ADO and RCP developed the intervention materials. DD and TF organized and conducted all of the assessments. DRL is the guarantor and accepts full responsibility for the conduct of the study and the integrity of the data. MJB and DRL are responsible for the accuracy of the preliminary data analysis. All authors have read and approved the final manuscript.

## Pre-publication history

The pre-publication history for this paper can be accessed here:

http://www.biomedcentral.com/1471-2458/10/652/prepub
